# Lifestyle Habits among Pregnant Women in Denmark during the First COVID-19 Lockdown Compared with a Historical Period—A Hospital-Based Cross-Sectional Study

**DOI:** 10.3390/ijerph18137128

**Published:** 2021-07-03

**Authors:** Hanne Kristine Hegaard, Ane Lilleøre Rom, Karl Bang Christensen, Lotte Broberg, Stinne Høgh, Cecilie Holm Christiansen, Nina Olsen Nathan, Mie Gaarskjaer de Wolff, Peter Damm

**Affiliations:** 1 Department of Obstetrics, Copenhagen University Hospital—Rigshospitalet, Blegdamsvej 9, 2100 Copenhagen, Denmark; ane.lilleoere.rom@regionh.dk (A.L.R.); lotte.broberg@regionh.dk (L.B.); stinne.hoegh@regionh.dk (S.H.); nina.olsen.nathan@regionh.dk (N.O.N.); mie.gaarskjaer.de.wolff.01@regionh.dk (M.G.d.W.); nis.peter.damm@regionh.dk (P.D.); 2 The Juliane Marie Centre, The Research Unit for Women’s and Children’s Health, Copenhagen University Hospital—Rigshospitalet, Blegdamsvej 9, 2100 Copenhagen, Denmark; cecilie.holm.christiansen.01@regionh.dk; 3 Department of Clinical Medicine, Faculty of Health and Medical Sciences, University of Copenhagen, Blegdamsvej 3B, 2200 Copenhagen, Denmark; 4 Research Unit of Gynecology and Obstetrics, Department of Clinical Research, University of Southern Denmark, J.B Winsløws Vej 4, 5000 Odense, Denmark; 5 Section of Biostatistics, Department of Public Health, University of Copenhagen, Østre Farimagsgade 5, 1353 K Copenhagen, Denmark; kach@sund.ku.dk; 6 Neurobiology Research Unit, Copenhagen University Hospital—Rigshospitalet, Blegdamsvej 9, 2100 Copenhagen, Denmark

**Keywords:** COVID-19, lockdown, pregnancy, lifestyle habits, pandemic, exercise, alcohol consumption, smoking

## Abstract

The first national lockdown in Denmark due to the COVID-19 pandemic was declared on 11 March 2020. From this date, national restrictions were imposed. We aimed to assess the potential influence of this first nationwide lockdown on exercise, alcohol consumption, and smoking in early pregnancy. Using a cross-sectional study based on routinely collected patient-reported data, we compared the lifestyle habits of women who were pregnant during the first phase of the pandemic (COVID-19 group) (*n* = 685) with those of women who were pregnant the year before (Historical group) (*n* = 787). We found a reduction in any exercise (PR = 0.91, 95% CI (0.84 to 0.99), in adherence to national recommendations of exercise (PR = 0.89, 95% CI (0.80 to 0.99), in cycling (15% vs. 28%, *p* < 0.0001), and swimming (0.3% vs. 3%, *p* = 0.0002) in the COVID-19 group compared with the Historical group. The prevalence of binge drinking was reduced in the COVID-19 group compared with the Historical group (PR = 0.80, 95% CI (0.68 to 0.93). In contrast, the prevalence of any weekly alcohol consumption and smoking cessation during pregnancy was similar between groups. Our findings indicate that national restrictions due to the COVID-19 pandemic influenced the lifestyle habits of pregnant women and should be addressed in antenatal counseling.

## 1. Introduction

On 11 March 2020, the coronavirus disease 2019 (COVID-19) outbreak was classified as a pandemic [1], and, on the same date, the first national lockdown was announced in Denmark [2], followed by numerous national restrictions. People were advised to work from home, to abstain from social events, and to keep a physical distance from people other than their closest relatives. Furthermore, child daycares, schools, educational institutions, and other public facilities such as libraries, museums, restaurants, non-essentials shops, and sports facilities were closed [2].

Changed circumstances such as the closure of sports and fitness facilities may result in reduced levels of exercise, and due to the closure of restaurants and bars, a decrease in the frequency of smoking and use of alcohol may occur [3]. Levels of smoking and alcohol consumption may also change with increased stress levels during a lockdown [4]. Stress has previously been associated with both abstinence and heavier drinking [5], and it has been reported that for some individuals, smoking serves as a perceived stress relief [6]. On the other hand, the pandemic may also be recognized as a “teachable moment”, leading to healthy changes in behavior [7].

Previous studies have shown that men and non-pregnant women changed their lifestyle habits during the COVID-19 pandemic [8,9,10,11,12,13,14,15,16,17]. Most studies reported decreased levels of exercise during the lockdown compared to before [8,11,18]. In contrast, an increase in walking has been described [8,9,12]. Prior studies have described both decreased and increased levels of alcohol consumption during the lockdown [13,14,15,16], and among women, the highest increase was seen in the age group 30–39 [13]. Among non-pregnant smokers, an increase in the number of daily cigarettes was reported during the COVID-19 pandemic [10,15,19,20]. However, a higher frequency of smoking cessation attempts has also been described [10,17,21].

A healthy lifestyle during pregnancy reduces the risk of adverse pregnancy outcomes [22,23,24,25,26,27,28,29]. Therefore, national and international health authorities recommend that pregnant women abstain from alcohol and smoking [30,31] and exercise according to the guidelines for pregnant women [22,23,32] Previous studies assessing the impact of the COVID-19 pandemic on exercise during pregnancy showed that most women exercised less during the lockdown, while only a few exercised more [33,34,35]. However, the existing research on exercise is scarce and based on relatively small study samples [33,34,35]. Thus far, no studies have investigated the impact of the COVID-19 pandemic on alcohol use and smoking during pregnancy. Knowledge about the potential changes in pregnant women’s lifestyle habits during a national lockdown is needed in antenatal care and counseling. Therefore, we aimed to assess the potential influence of the first nationwide lockdown in Denmark due to the COVID-19 pandemic on the level and type of exercise, alcohol consumption, and smoking status in early pregnancy.

## 2. Materials and Methods

We performed a cross-sectional study comparing a group of women who were pregnant during the first COVID-19 national lockdown (COVID-19 group) with a reference group of women who were pregnant in the same period the year before (Historical group). We used patient-reported clinical data collected consecutively since 2012 at the Department of Obstetrics, Copenhagen University Hospital—Rigshospitalet, Denmark. The Department serves as a tertiary referral center and is the primary birth facility of central Copenhagen, with more than 5000 births annually. All pregnant women who schedule their first-trimester ultrasound scan routinely receive an email invitation to fill in an electronic questionnaire available in Danish or English. The scan is part of the national prenatal screening program and has ≥90% participation [36]. The questionnaire includes items on maternal reproductive history, lifestyle habits during pregnancy, maternal morbidity, and socio-demographic characteristics. The information is routinely transferred to medical records for use during antenatal care. A previous study showed that women filled in the questionnaire at a mean of 10.2 (SD = 2.1) weeks of gestation [37].

**Figure 1:**
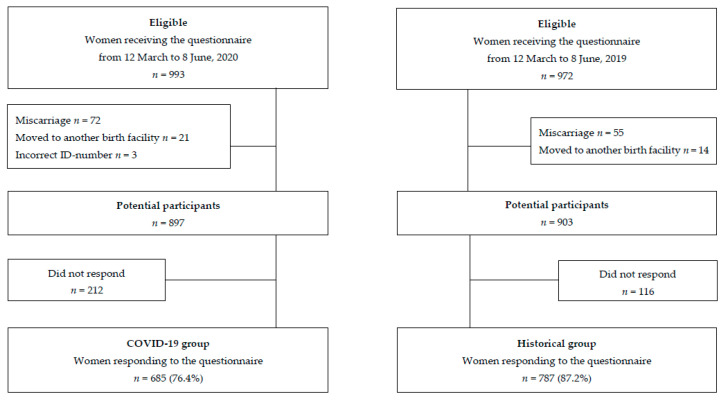
Flowchart of the study population.

Pregnant women who received a link to the questionnaire from 12 March to 8 June 2020 (*n* = 993) or in the corresponding period in 2019 (*n* = 972) were eligible. After excluding women who had a miscarriage, moved to another birth facility, or had an incorrect identification (ID) number, 685/897 (76.4%) women in the COVID-19 group and 787/903 (87.2%) in the Historical group responded (Figure 1). The date 8 June 2020 was set as the end date for the COVID-19 group because from this date, sports facilities, including public swimming pools and fitness and sports centers, were re-opened in Denmark [35]. Women who received a link to the questionnaire on June 8 were included if they responded before 18 June in both 2019 and 2020. Women responding after 18 June in the COVID-19 group (*n* = 10) and in the Historical group (*n* = 7) were categorized as non-responders.

### 2.1. Outcomes

#### 2.1.1. Exercise

In the questionnaire, the question on exercise was phrased as follows: Do you engage in any exercise at present (yes/no)? A positive response prompted a question about specific types of exercise and duration (hours/week). The 10 specific types included running, strength training, yoga, cycling (also for transport to and from work), brisk walking, spinning, fitness, swimming, aquatic exercise, horseback riding, and “other type of exercise”. Responses to “other type of exercise” could be specified in free text. Any exercise was defined as a positive answer to any exercise type, and the recommended level of exercise was defined as ≥3.5 h of exercise per week in accordance with national recommendations [32].

#### 2.1.2. Alcohol Consumption

The question on weekly alcohol consumption was phrased as follows: How many drinks do you currently have per week now that you are pregnant (one drink equivalent to one bottle of beer, one glass of wine, or 4 cl. spirits)? The question on binge drinking was phrased as follows: How many episodes have you had of drinking 5 or more units on a single occasion during pregnancy*?* In the questionnaire, it was emphasized that this might also include the period before the pregnancy was recognized. Weekly alcohol consumption was categorized as 0 units versus >0 units/week. The answers regarding binge drinking were classified by (yes/no) and by the number of episodes (0, 1, ≥2, or do not know/remember).

#### 2.1.3. Smoking

The first question on smoking was phrased as follows: Did you smoke before you became pregnant? A positive answer prompted the following question: Are you a smoker now? Using the answers to these two questions, we categorized smoking status during pregnancy as smokers/non-smokers/quitters. Smokers were defined by responding “yes” to both questions, non-smokers by responding “no” to both questions and quitters were defined as women who responded yes to the first question and no to the second.

### 2.2. Covariates

The questionnaire also contained patient-reported data on the selected covariates: age, parity, highest obtained educational level, pre-pregnancy body mass index (BMI), Danish language skills, previous miscarriage, assisted reproductive technology chronic disorder, previous contact with a psychiatrist and/or self-reported psychiatric condition, cohabitation status, occupation, and degree of pregnancy planning. The included covariates were categorized as shown in Table 1.

### 2.3. Ethical Approval

The study was approved by the National Data Protection Agency (file no- RH-2016-202. I-suite no.:04778, December 2017). Patient consent was waived due to the Danish Patient Safety Authorities having granted permission to disclose patient information from medical records for research use (file no. 31-1521-399, 10 June 2020). Permission was obtained from Medical Records Research, Health Research and Innovation Center for Regional Development, The Capital Region of Denmark (file no. R-20066400, 30 November 2020).

### 2.4. Statistical Analyses

The distribution of the selected covariates and the included outcomes were assessed descriptively and reported as mean and standard deviation (SD) for continuous data and as frequency (*n*) and proportion (%) for categorical data. The distributions were compared across groups using t-tests for continuous data and chi-squared tests for categorical data.

Any exercise (yes/no), adherence to the national recommendations of exercise (yes/no), and binge drinking (yes/no) were compared across groups using log-binomial regression models [38], estimated prevalence ratios (PRs), and corresponding 95% confidence intervals (CI). We reported crude estimates and estimates adjusted for potential confounders chosen a priori based on existing evidence [39,40,41,42,43,44,45]. Two adjusted models were applied: regarding measures of exercise, in the simple adjusted model, we adjusted for age, parity, and highest obtained educational level, whereas in the fully adjusted model, we additionally adjusted for BMI, Danish language skills, previous miscarriage, assisted reproductive technology, chronic disorder, and previous contact with a psychiatrist and/or self-reported psychiatric condition. Regarding binge drinking, we adjusted for age, parity, and highest obtained educational level in the simple model; in the fully adjusted model, additional adjustment was made for assisted reproductive technology, pregnancy planning, previous miscarriage and previous contact with a psychiatrist and/or a self-reported psychiatric condition. In the primary analyses on binge drinking, we excluded women who responded that they “did not remember” whether they had had binge drinking episodes during early pregnancy. In the sensitivity analyses, we included these women in the group who had episodes of binge drinking and (2) included them in the group of women who had no episodes of binge drinking to test the robustness of the estimates.

Furthermore, we compared the prevalence of women who reported smoking cessation during pregnancy across the two groups. No adjusted analyses are reported for this outcome due to the small sample size. In all adjusted analyses, missing data were imputed using multiple imputations by chained equations (20 imputations) under the assumption of missing at random. The maternal age category was used in the imputation model. Participation in different types of exercise activities was compared across groups graphically using clustered bar charts, and group differences were tested using chi-squared tests. *p*-values are reported along with *p*-values adjusted for multiple testing using the Benjamini–Hochberg correction, adjusting the false discovery rate (FDR) at 5% [46]. The statistical analyses were performed using SAS 9.4 (SAS Institute Inc., Cary, NC, USA).

## 3. Results

No differences were found in maternal characteristics between the COVID-19 group and the Historical group, except for reporting of previous contact with a psychiatrist and/or having a psychiatric condition; in the COVID-19 group, a total of 11% was reported compared with 7% in the Historical group (*p* = 0.0164) (Table 1).

### 3.1. Exercise

The prevalence of any exercise was lower in the COVID-19 group (59%) compared with the Historical group (63%) (PR = 0.90, 95% CI (0.80 to 1.01)) (Table 2). After a simple adjustment, the prevalence was 7% lower in the COVID-19 group (PR = 0.93, 95% CI (0.86 to 1.01). After full adjustment, the prevalence was significantly reduced by 9% compared with the Historical group (PR = 0.91, 95% CI (0.84 to 0.99) (Table 2). Similarly, the prevalence of women who adhered to the recommended level of weekly exercise was lower in the COVID-19 group (43%) than in the Historical group (48%) (PR = 0.90, 95% CI (0.80 to 1.01)) (Table 2). After simple as well as full adjustment, a reduction of 11% was found (PR = 0.89, 95% CI (0.80 to 0.99) (Table 2).

**Figure 2:**
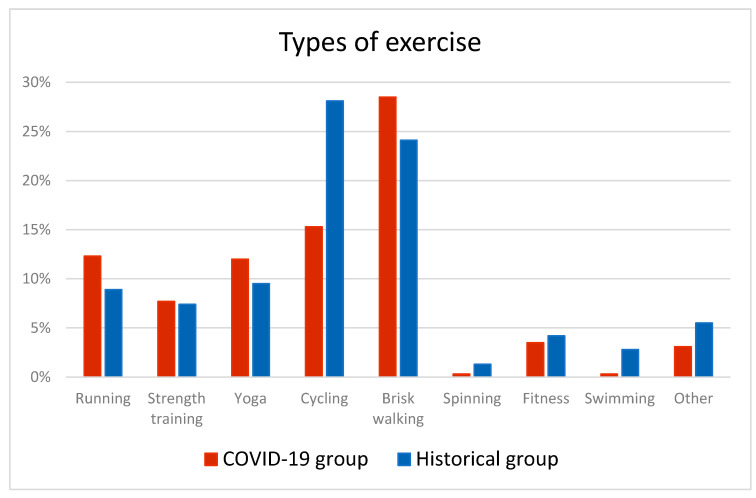
The distribution of types of exercise in the COVID-19 group and in the Historical group. Differences across groups were significant for cycling (FDR < 0.0001) and swimming (FDR = 0.0002) after correction for multiple testing.

In the COVID-19 group, the five most reported types of exercise were brisk walking (28%), cycling (15%), running (12%), yoga (12%), and strength training (8%). In the Historical group, the five most reported types of exercise were cycling (28%), brisk walking (24%), yoga (10%), running (9%), and strength training (7%) (Figure 2). In the COVID-19 group, only a few women were engaged in swimming (0.3%), spinning (0.3%), and fitness (4%), compared with 3%, 1%, and 4% in the Historical group, respectively (Figure 2). The proportion of women reporting all other types of exercises was 3% in the COVID-19 group and 5% in the Historical group. Engagement in horseback riding and aquatic exercise was low (*n* = 3) and, therefore, included in the category “other type of exercise” (Figure 2). Differences across groups were significant for cycling (*p* < 0.0001; FDR < 0.0001) and swimming (*p* = 0.0002; FDR = 0.0007) only. Differences across groups were significant for cycling (FDR < 0.0001) and swimming (FDR = 0.0002) after correction for multiple testing.

### 3.2. Alcohol Consumption

The prevalence of alcohol consumption on a weekly basis was similar across groups, with 1% of women reporting an intake of >0 units/week in both groups (data not shown). In the COVID-19 group, 23% (161) of the women reported episodes of binge drinking during early pregnancy, while this was the case for 30% (239) of the women in the Historical group. We found a 24% reduction in episodes of binge drinking in the COVID-19 group compared with the Historical group (PR 0.76, 95% CI (0.64–0.89) (Table 2), and after adjustment (simple and full), the prevalence was reduced by approximately 20% (PR = 0.79, 95% CI (0.67 to 0.93) and PR = 0.80, 95% CI (0.68 to 0.93), respectively) (Table 2). Women who responded “do not know/remember” were not included in these analyses. Including them in either the group of women who had episodes of binge drinking or in the group of women who had no episodes of binge drinking did not change the results (data not shown).

**Figure 3:**
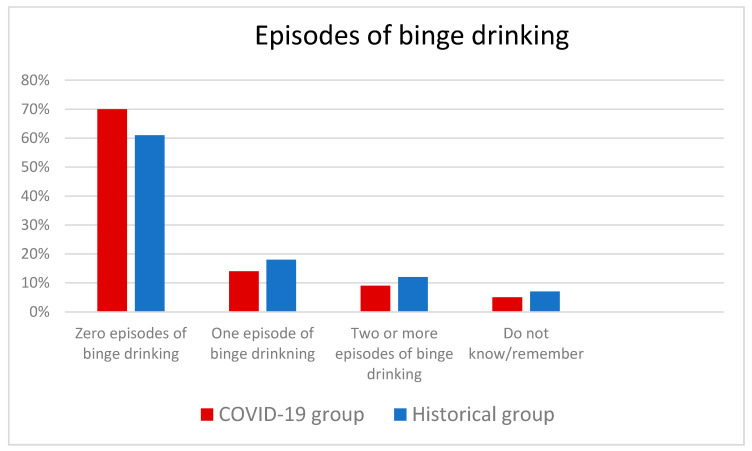
The distribution of binge drinking episodes in the COVID-19 group and the Historical group. The difference across the groups was significant (*p* = 0.004).

Among the women who reported episodes of binge drinking in the COVID-19 group, 14% reported one episode and 9% reported two or more episodes compared with 18% and 12%, respectively, in the Historical group. The difference across the groups was significant (*p* = 0.004) (Figure 3).

### 3.3. Smoking

The prevalence of smokers, non-smokers, and quitters was similar across the two groups (*p* = 0.868) (Table 1). When comparing the prevalence of women who reported smoking cessation during pregnancy across the two groups, no difference was found (PR = 1.00, 95% CI (0.91 to 1.09)) (Table 2). Due to the small number of smokers, it was not possible to perform any adjusted analyses.

## 4. Discussion

Using consecutively collected data, we found that the prevalence of any exercise, adherence to the recommended level of exercise, and binge drinking was significantly lower during the lockdown compared with the year before. The prevalence of any weekly alcohol consumption and smoking cessation was, however, similar. Furthermore, we found significant changes in specific types of exercise—e.g., the proportion of women engaged in cycling (also for transportation to and from work) was halved during the lockdown compared with the year before.

Previous studies have demonstrated that the majority of pregnant women reduced their level of exercise during the lockdown compared to before [33,34,35], while only a smaller group increased their level of exercise [33,34]. It is not possible directly to compare our results with results from previous studies since these assessed the level of exercise before and during the lockdown within the same group of women [33,34,35]. It is well known that the level of exercise decreases as the pregnancy progresses [40,47], and the results from the previous studies [33,34,35] may partly be explained by normal changes during pregnancy rather than by a change due to the COVID-19 pandemic [33,34,35]. Likewise, studies among non-pregnant women demonstrated decreased exercise levels during the lockdown compared to before [8,48,49]. In the present study, a lower level of exercise was expected in the COVID-19 group given the restrictions imposed on indoor exercise facilities and the greater extent of working from home, most likely resulting in lower levels of, e.g., cycling to and from work. In line with this, a previous study found that non-pregnant women were less physically active during the first lockdown due to less transportation to and from work [11]. In addition, prior to the lockdown, the average female citizen in Copenhagen was found to transport herself 2.6 km by bicycle daily [50]. In studies of non-pregnant persons, brisk walking was more frequently reported during the lockdown [9,49]. In our study, the reported increase in brisk walking was not statistically significant.

The prevalence of women who met the recommended level of exercise during the lockdown was lower than that in the Historical group (43% vs. 48%). However, the prevalence of pregnant women who adhered to the recommended level of exercise during the lockdown still remains high compared with previous studies performed before the COVID-19 pandemic [51,52,53]. This may partly be explained by the high proportion of well-educated women in our population. A positive association between high education level and level of exercise is well documented [40,47,51,53].

The reduced level of exercise during the lockdown was smaller than we expected but still of concern since it theoretically may increase the risk of maternal complications such as preeclampsia, gestational diabetes, increased gestational weight, and mental health complications [22,23] Further, a reduced level of exercise during the lockdown may increase the risk of giving birth to a macrosomic infant, but may not impact other neonatal outcomes [54].

The impact on mental health is particularly relevant since it has been described that pregnant women have been challenged by mental health complications during the lockdown [55,56].

During pregnancy, the weekly use of alcohol was reported to be very low (1%) in both groups. Since an increase in alcohol use has been found in non-pregnant women in the same age group and among postpartum women during the lockdown, it is positive that no increase was found among pregnant women in our study. In total, 23% in the COVID-19 group and 30% in the Historical group reported binge drinking during early pregnancy. These findings are in line with the prevalence described in previous Danish studies [41,57] and with the reported prevalence during the lockdown among Norwegian non-pregnant women aged 25–29 years (25%), but are higher than the reported prevalence among women aged 30–39 years (9%) [13]. However, the women in the COVID-19 group reported significantly fewer episodes of binge drinking compared with the Historical group. The explanation for this may be found in the restrictions as well as in social distancing. Alcohol is harmful to the fetus, and a decrease in the prevalence of binge drink in the COVID-19 group may positively affect perinatal outcomes [58].

As seen in other Danish studies, the prevalence of pregnant smokers was low in both groups [43]. However, no difference in smoking cessation was found between groups. In line with this, studies among non-pregnant persons during the COVID-19 pandemic have shown an increase in smoking cessation attempts and a higher motivation towards smoking cessation [10,21]. Specific reasons given for smoking cessation were to live a healthier life, to have healthier lungs, to be healthier, and to help in recovery from a potential coronavirus infection [10,17]. Conversely, a lower cessation rate could also have been anticipated in the COVID-19 group as women experienced the COVID-19 pandemic as stressful [4]. Previous studies have shown that female smokers more often use smoking to cope with stress compared with male smokers [59].

A strength of our study was the use of routinely collected data on lifestyle during pregnancy. These self-reported data have been collected consecutively since 2012, allowing us to use valid data on outcomes and selected covariates. Furthermore, we could compare data collected during the lockdown with corresponding data from the year before, which reduced the risk of bias due to seasonal trends.

We found that the non-responding rate was higher in the COVID-19 group than in the Historical group. We do not have information on non-responders, but it is well known that responders typically are of higher socio-economic status and more healthy than non-responders [60]. If the COVID-19 group would then include more healthy responders, more women in the COVID-19 group may then have exercised, and fewer may have binge-drinked compared to the Historical group, potentially underestimating the true effect of COVID-19 on exercise, and overestimating the true effect on binge-drinking. Further, the difference in response rate may introduce differences in maternal characteristics between the two groups and thus introduce confounding. To reduce this risk, we adjusted for several potential confounders, including, e.g., educational level, which may to some extent act as a proxy for lifestyle behavior [39,43].

In the analyses of any exercise, the level of significance changed after full adjustment (model 2). The point estimates were, however, similar and the confidence intervals were considerably overlapping. We cannot be sure whether a change in the level of significance was due to over-adjustment or to smaller residual variation due to better confounder control.

We found a significantly higher proportion of pregnant women with previous contact with a psychiatrist and/or having a psychiatric condition in the COVID-19 group than in the Historical group. This difference could potentially bias the estimates. However, adjustment for previous contact with a psychiatrist and/or having a psychiatric condition, was made in all the adjusted analyses (model 2).

The assessment of exercise relied on self-reporting, which, compared to objective measures, entails a risk of misclassification [61]. A previous study among pregnant women found that pregnant women who exercise more than 150 min weekly under-reported their level of exercise. In contrast, inactive pregnant women over-reported the level [62]. However, the item used to measure exercise was a modified version of the Minnesota Leisure Time Physical Activity Questionnaire, which is validated, although not for pregnant women [63]. A limitation of the questionnaire is that it does not assess any home-based training, which would have been relevant during the lockdown, as we expected some women to be engaged in online training at home. The proportion of women engaged in yoga and strength training was similar across the two groups, and since training facilities were closed, it may have been home-based or outdoor training. Additionally, the use of self-administered questionnaires is known to return an underestimation of alcohol consumption during pregnancy in comparison with data diaries or interviews [64,65]. However, a strength is that we collected information about binge drinking at around 10 gestational weeks, which has been shown to yield more precise estimates than later in pregnancy [66]. Similarly, smoking can be underestimated compared with objective measurements. However, a Swedish study showed a high correlation between serum cotinine and self-reported smoking status during pregnancy [67].

During the pandemic, there has been an increased focus on healthy lifestyle, since it has been debated, whether e.g., smoking and obesity could increase the risk of the severity of COVID-19 infection [68,69]. This could potentially increase the existing risk of underreporting unhealthy behaviors [64,65,70] during the pandemic compared to before. If this was the case, pregnant women in the COVID-19 group may underreport alcohol consumption and smoking to a higher degree than pregnant women in the Historical group, which may potentially overestimate our findings regarding these outcomes. Conversely, a potential overreporting of healthy behavior i.e., exercise in the COVID-19 group may underestimate the effect on exercise. We do, however, not know whether the pandemic had influenced the reporting of lifestyle among pregnant women.

Finally, most of the women were Danish speaking, cohabiting, well educated, employed, and had a normal BMI. The results from this study should, therefore, be interpreted with caution when transferred to other populations.

Our findings indicate that it is important to address lifestyle habits as part of antenatal counseling when a national lockdown is initiated. To maintain the recommended level of exercise during pregnancy, women should be advised to perform types of exercise that are feasible during a lockdown [70]. This is especially relevant when women are required to work from home and thereby reduce their level of physical activity linked to commuting, or if the training facilities they usually attend are closed. Brisk walking and yoga seem to be feasible types of exercise for pregnant women in a lockdown situation. To improve exercise among pregnant women, it may also be relevant for maternity care providers to offer pregnant women free online exercise webinars.

This study examines the impact of the COVID-19 pandemic on lifestyles habits including exercise, alcohol consumption, and smoking. Future studies should address whether differences in lifestyle behaviors between groups impact maternal and fetal health outcomes, e.g., preeclampsia, gestational diabetes, preterm delivery, gestational weight gain, and birth weight [22,23,24,25,26,27,28,29] in larger epidemiological studies. In addition, a potential impact on maternal and fetal health after birth should be assessed.

## 5. Conclusions

Women who were pregnant during the first national lockdown in Denmark due to the COVID-19 pandemic changed their lifestyle habits in early pregnancy, with a lower level of exercise and a lower level of binge drinking compared to women who were pregnant the year before.

## Figures and Tables

**Table 1:** Characteristics of the COVID-19 group and the Historical group.

	COVID-19 Group	Historical Group	*p-*Value
	*n* (%)	*n* (%)	
Total (N = 1472)	685 (47)	787 (53)	
Characteristics			
Age (years)			0.0633
18–24	27 (4)	20 (3)	
25–29	189 (28)	257 (33)	
30–34	307 (45)	317 (40)	
≥35	161 (24)	193 (25)	
Missing	1 (0)	0	
Mean (±SD)	31.5 (±4.2)	31.6 (±4.4)	0.7092
Parity			0.5460
Nullipara	452 (66)	531 (67)	
Multipara	233 (34)	256 (33)	
Highest obtained educational level			0.6330
Higher degree	413 (60)	472 (60)	
Intermediate degree (3–4 years)	39 (6)	43 (5)	
Short degree (1–2 years)	14 (2)	22 (3)	
Technical degree	186 (27)	195 (25)	
Compulsory education	29 (4)	42 (5)	
Missing	4 (1)	13 (2)	
Body Mass Index (BMI)(kg/m^2^)			0.5782
Underweight (<18.5)	24 (4)	38 (5)	
Normal (18.5–24.9)	511 (75)	578 (73)	
Overweight (25–29.9)	95 (14)	105 (13)	
Obese (≥30)	36 (5)	36 (5)	
Missing	19 (3)	30 (4)	
Mean (±SD)	22.8 (±3.9)	22.8 (±3.8)	0.7300
Danish language skills			0.8691
Yes	638 (93)	737 (94)	
Missing	11 (2)	10 (1)	
Previous miscarriage			0.3213
Yes	196 (29)	207 (26)	
Assisted reproductive technology (ART)			0.1634
Yes	90 (13)	123 (16)	
Missing	1 (0)	5 (1)	
Chronic disorder			0.3377
Yes	191 (28)	202 (26)	
Previous contact with a psychiatrist and/or self-reported psychiatric condition			
Yes	72 (11)	55 (7)	0.0164
Cohabitation			0.0659
Yes	640 (93)	713 (91)	
Occupation			0.1775
Employed	508 (74)	615 (78)	
Unemployed	47 (7)	39 (5)	
Student	85 (12)	86 (11)	
Other	47 (7)	39 (5)	
Missing	3 (0)	8 (1)	
Pregnancy planning			0.9217
Very planned	335 (49)	371 (47)	
Fairly planned	177 (26)	208 (26)	
Neither planned nor unplanned	117 (17)	140 (18)	
Fairly unplanned	25 (4)	34 (4)	
Very unplanned	24 (4)	25 (3)	
Missing	7 (1)	9 (1)	
Smoking status during pregnancy			0.8680
Smokers	5 (1)	6 (1)	
Non-smokers	613 (89)	698 (89)	
Quitters	64 (9)	80 (10)	
Missing	3 (0)	3 (0)	

Percentages may not sum to 100 due to rounding of decimals.

**Table 2:** Prevalence ratios (PRs) and associated 95% confidence intervals (CI) of exercise, binge drinking, and smoking cessation in the COVID-19 group compared with the Historical group.

	Group	N	Prevalence of Outcome (%) *n*	Crude		Adjusted			
				PR	(95% CI)	Model 1		Model 2	
						PR	(95% CI)	PR	(95% CI)
Any Exercise *	COVID-19	681	59 (406)	0.95	(0.87 to 1.03)	0.93	(0.86 to 1.01)	0.91	(0.84 to 0.99)
	Historical	783	63 (492)	1.00	-	1.00	-	1.00	-
Adherence to recommended level of exercise (≥3.5 h/week)	COVID-19	685	43 (294)	0.90	(0.80 to 1.01)	0.89	(0.80 to 0.99)	0.89	(0.80 to 0.99)
	Historical	787	48 (376)	1.00	-	1.00	-	1.00	-
Binge drinking **	COVID-19	641	23 (161)	0.76	(0.64 to 0.89)	0.79	(0.67 to 0.93)	0.80	(0.68 to 0.93)
	Historical	719	30 (239)	1.00	-	1.00	-		-
Smoking cessation	COVID-19	69	9 (64)	1.00	(0.91 to 1.09)	-	-	-	-
	Historical	86	10 (80)	1.00	-	-	-	-	-

*: Model 1: adjusted for maternal age, parity, educational level. Model 2: adjusted for maternal age, parity, educational level, Body Mass Index, Danish language skills, previous miscarriage, Assisted Reproductive Technology, chronic disorders, and previous contact to a psychiatrist and/or self-reported psychiatric condition. **: Model 1: adjusted for maternal age, parity, educational level. Model 2: adjusted for maternal age, parity, educational level, Assisted Reproductive Technology, pregnancy planning, previous miscarriage, and previous contact to a psychiatrist and/or self-reported psychiatric condition.

## Data Availability

The data are not publicly available due to limitations in the permission granted from Danish Patient Safety Authorities.
